# Dataset on elemental composition of soils and plants under long-term application of mineral and organic fertilizers on gray forest soils in Vladimir region, Russia

**DOI:** 10.1016/j.dib.2024.110057

**Published:** 2024-01-11

**Authors:** A.D. Kotelnikova, T.I. Borisochkina, K.A. Kolchanova, M.A. Shishkin, F.S. Egorov, V.V. Okorkov, O.B. Rogova

**Affiliations:** a FRC V.V. Dokuchaev Soil Science Institute, 7 Pyzhevsky Lane building 2, Moscow 119017, Russian Federation; b State Scientific Institution Vladimir Research Institute of Agriculture, Vladimirskaya oblast, Suzdalskiy rayon, p. Noviy 601261, Russian Federation

**Keywords:** Trace elements, Macro elements, Rare earth elements, Soil organic carbon, Chemical properties, Agronomy

## Abstract

Long-term application of organic and mineral fertilizers can lead to changes in the elemental composition of agroecosystem components. Both the levels of nutrients and potentially toxic elements can change, as can the potential for these elements to be available to plants through changes in soil properties. Soil and plant samples of two species (pea *Pisum sativum* L. and oat *Avena sativa* L.) were collected from plots of a long-term field experiment on the application of mineral and organic fertilizers and their combinations to gray forest soils in the Vladimir region, Russia. Soil samples from the 0-20 and 20-40 cm layers were subjected to acid digestion to determine total element content. Mobile forms of elements were extracted from topsoil samples using acetate-ammonium buffer (pH 4.8). Sample preparation of pea and oat plant organs (stems, leaves, pods/ears) included sample digestion in a microwave sample digestion system ETHOS EASY (Milestone, Italy). The elemental composition of the samples was determined by inductively coupled plasma optical emission spectrometry (ICP-OES) using Agilent 5800 ICP-OES (Agilent Technologies, USA). The dataset includes concentration data for 34 elements, including rare earth elements, in these samples collected in 2021. The dataset also contains general agrochemical characteristics of soils of the experimental groups: pH of water and salt suspension, organic carbon content, mobile forms of phosphorus. The data can be valuable to researchers developing fertilizer application systems and modeling changes in the elemental composition of agroecosystems.

Specifications Table

**Table:** 

Subject	Agricultural Sciences: Agronomy and Crop Science, Soil Science
Specific subject area	Elemental composition of agrocenosis components - soils and plants - in a long-term field experiment
Data format	Raw data
Type of data	Table
Data collection	Data on elemental composition of soil and plant samples were obtained by inductively coupled plasma optical emission spectrometry (ICP-OES) using Agilent 5800 ICP-OES (Agilent Technologies, USA). Acid digestion of the samples was carried out beforehand. Data on soil pH-value were obtained potentiometrically for aqueous (H_2_O) and saline (KCl) suspension at a ratio of 1:2.5 using Sartorius PB-11 (Sartorius AG, Finland). Soil organic carbon content was determined by the Tyurin's method with photometric termination, using a UNICO-1201 spectrophotometer (UNICO, USA). The content of mobile phosphorus was also determined photometrically.
Data source location	Country: Russia Region: Vladimir region, Suzdal GPS coordinates of the experimental field: 56°25′02" N 40°25′25" E
Data accessibility	Repository name: Mendeley Data Data identification number: 10.17632/3cygrjjtf3.1 Direct URL to data: https://data.mendeley.com/datasets/3cygrjjtf3/1

## 1 Value of the Data

• The dataset is of interest to soil scientists, agronomists and agroecologists as it provides information on the concentrations of a wide range of elements in soils and associated plants from a unique long-term field experiment in the European part of Russia on soils widely used in agriculture.
• The dataset can be useful to researchers involved in developing different fertilizer application systems and modeling the effects of such systems on soil fertility and crop yields.
• The data can be used to develop and justify fertilizer application rates.
• The special value of the dataset lies in the data on the concentration of rare earth elements in soils and plants. Such data are still limited and additional information is needed for different areas, soil types and plant species.

## 2 Data Description

**Fig. 1:**
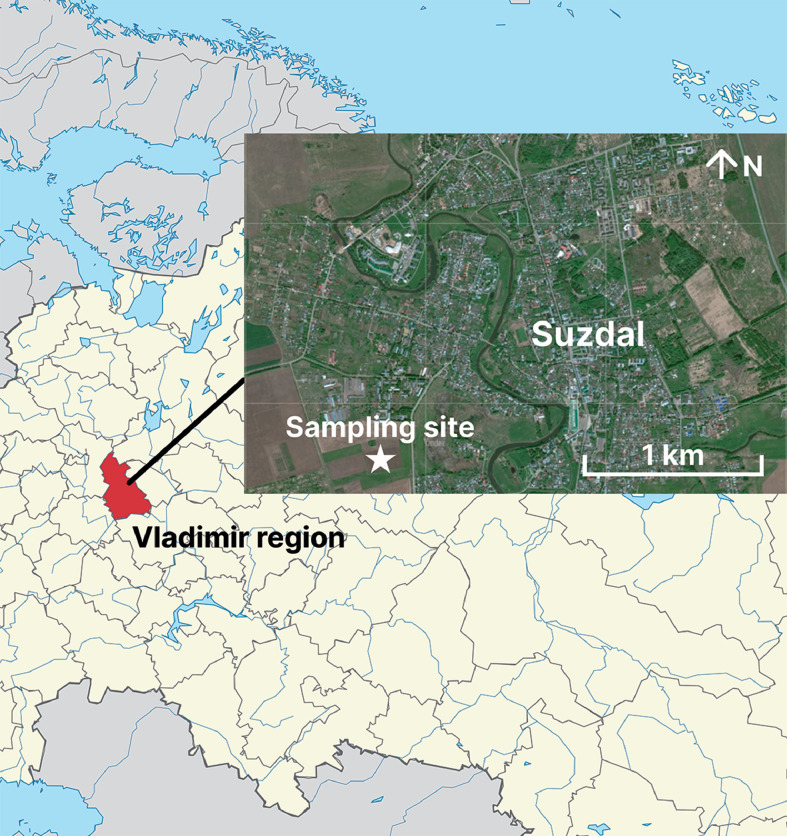
Location of the sampling site.

The dataset [1] contains data on the elemental composition of agrocenosis components – soils and plants (pea *Pisum sativum* L. and oat *Avena sativa* L.) – samples of which were collected from plots of a long-term field experiment on gray forest heavy loamy soil in July 2021. Samples for data acquisition were collected near the city of Suzdal, Vladimir region, Russia (Fig. 1). According to WRB classification (World Reference Base for Soil Resources) soil of the experimental field – Luvic Retic Greyzemic Phaeozem (Loamic, Aric) [2]. Long-term field experiment on plots from which samples were taken is included in the geographic network of experiments with fertilizers (Geonet) in Russia under the title “Effect of fertilizer systems in crop rotation on crop productivity, fertility of gray forest soil and its ecological state” [3]. The experiment was set up in 1991 and is still being maintained at the present time [4]. Samples were taken at the beginning of the fourth rotation of the crop rotation, which includes the following crops: annual grasses (pea-oat mixture); winter wheat; oats with grass sowing; first year grasses; second year grasses; winter wheat; barley. Data were obtained for each of the 9 experimental groups of the long-term field experiment, including applications of both mineral and organic fertilizers, as well as their combinations (Table 1). Table 1 summarizes the designations used for the experimental groups in the dataset and their characteristics. Phosphorus-potassium fertilizers were applied under the main cultivation, nitrogen fertilizers were applied in the pre-sowing crop for annual grasses and spring cereals, in the top dressing of winter crops and perennial grasses. Cattle manure was applied to winter wheat after a pea-oat mixture.

**Table 1:** Description of the experimental groups of the long-term field experiment used for sampling to obtain the dataset.

Experimental group	Description of the experimental group
Control	Control group, soil to which nothing was applied
Background	Soil limed at the beginning of the first rotation of the crop rotation. Background for experimental groups with fertilizer application, which were also limed
PK	Phosphorus and potassium fertilizers were applied to the soil in one dose per rotation: double superphosphate (P_2_O_5_ single dose 340 kg/ha), potassium salt (KCl 360 kg/ha)
1NPK	Nitrogen, phosphorus and potassium fertilizers were applied to the soil in one dose per rotation: double superphosphate (P_2_O_5_ single dose 340 kg/ha per rotation), potassium salt (KCl 360 kg/ha)
2NPK	Nitrogen, phosphorus and potassium fertilizers were applied to the soil in double dose per rotation: ammonium nitrate (NH_4_NO_3_ 680 kg/ha per rotation), double superphosphate (P_2_O_5_ 680 kg/ha), potassium salt (KCl 720kg/ha)
Manure	Cattle manure was applied to the soil (60 t/ha, 6.2 kg N, 3.2 kg P_2_O_5_, 6.1 kg K_2_O in 1 t of manure)
Manure+PK	Cattle manure was applied to the soil (60 t/ha) in combination with phosphorus and potassium fertilizers in one dose per rotation
Manure+1NPK	Cattle manure was applied to the soil (60 t/ha) in combination with nitrogen (340 kg/ha), phosphorus (340 kg/ha) and potassium fertilizers in one dose per rotation
Manure+2NPK	Cattle manure was applied to the soil (60 t/ha) in combination with nitrogen, phosphorus and potassium fertilizers in double dose per rotation

In the form of a table, a single file contains concentration data for 34 elements obtained using the inductively coupled plasma optical emission spectrometry (ICP-OES). Dataset is available in the repository (https://data.mendeley.com/datasets/3cygrjjtf3/10). The dataset presents concentrations of the following elements: Na, P, Ca, K, Mg, Fe, Al, Li, Cr, Mn, Co, Ni, Cu, Zn, Sr, Cd, Ba, Pb and rare earth elements. Rare earth elements represent a separate group in the dataset and include La, Ce, Pr, Nd, Sm, Eu, Gd, Tb, Dy, Ho, Er, Tm, Yb, Lu, Sc, Y. Data reporting units are mg/kg, or mg/g. Data on the total content of elements in soil (0-20 and 20-40 cm layer), content of mobile forms of elements in soil (0-20 cm layer), content of elements in separate organs (leaves, stems, fruits) of two plant species - pea and oat, and data on the content in above-ground organs of plants as a whole, obtained taking into account the mass fraction of each organ in the plant, on separate sheets of Excel file are presented. The dataset also includes a number of physico-chemical characteristics of soils: pH of water and saline suspension, organic carbon content, content of mobile forms of phosphorus. For each experimental group, three mixed samples were analyzed; accordingly, the dataset presents data for each repetition (sample). The table file contains 11 sheets (Table 2).

**Table 2:** Contents of the dataset table.

Sheet number	Sheet name in the table	Table sheet contents
1	1_Soil physicochemical data	Physico-chemical characteristics of soils of experimental groups in 0-20 and 20-40 cm layer: pH of water and salt suspension, organic carbon content (%), content of mobile forms of phosphorus (mg/kg)
2	2_Soil total	Total content of elements in soils of experimental groups in layers 0-20 and 20-40 cm (mg/kg or mg/g)
3	3_Soil mobile	Content of mobile forms of elements in soils of experimental groups in 0-20 cm layer (mg/kg)
4	4_Pea steams	Element content in pea steams in experimental groups (mg/kg)
5	5_Pea leaves	Element content in pea leaves in experimental groups (mg/kg)
6	6_Pea pods	Element content in pea pods in experimental groups (mg/kg)
7	7_Pea aboveground	Element content in pea aboveground organs in experimental groups (mg/kg)
8	8_Oat steams	Element content in oat steams in experimental groups (mg/kg)
9	9_Oat leaves	Element content in oat leaves in experimental groups (mg/kg)
10	10_Oat ears	Element content in oat ears in experimental groups (mg/kg)
11	11_Oat aboveground	Element content in oat aboveground organs in experimental groups (mg/kg)

## 3 Experimental Design, Materials and Methods

Soil samples were collected from 0-20 and 20-40 cm layers. One mixed sample was formed from three individual samples. Three such mixed samples were taken from each experimental plot. Plant samples were also collected in triplicate for each experimental group. The area of one plot is 100 m^2^.

Soil samples were dried at room temperature to air-dry condition, ground in a porcelain mortar and sieved through a sieve with 1 mm hole diameter according to the Russian State standard [5] and then stored in plastic bags. Plant samples were dried at room temperature in a ventilated unlit room. Plant samples were divided into individual organs - leaves, stems, pods or ears, and the mass of each part was recorded. The samples were then pulverized and stored in plastic bags until analysis.

Water (H_2_O) and salt (1 M KCl) pH of soil samples were determined potentiometrically (Sartorius PB-11, Sartorius AG, Finland) at a suspension with solution to soil sample ratio of 1:2.5 [6]. Soil organic carbon content was determined by wet dichromate oxidation according to the modified Tyurin's method with photometric ending using a UNICO-1201 spectrophotometer (UNICO, USA) [7]. The content of exchangeable phosphorus was determined photometrically according to the Kirsanov method [8].

Dry soil and plant samples were subjected to sample preparation prior to elemental analysis by ICP-OES method. To determine the total content of elements in soil samples, complete acid digestion of samples was carried out with a mixture of nitric, perchloric and hydrofluoric acids. A soil sample (0.2 g) is placed in a Teflon beaker (50 mL volume), moistened with a few drops of distilled water, 2 mL of 37 % H_2_O_2_ and 2 mL of water are added. The beakers are placed on a stove and boiled until evaporated. Then 1 mL of 65% HNO_3_, 1 mL of 65% HClO₄ and 6 mL of 45% HF are added to the Teflon beakers. The beakers are placed on a stove, covered with a Teflon lid, brought to a boil and heated for 30 min. Then the lids are removed and evaporated until the appearance of intense white vapors, remove the cups from the stove, cooled, washed the walls with a small amount of distilled water and further evaporated to wet salts. Then 4 mL of 37% HCl and 0.4 mL of 0.1 M H_3_BO_3_ are added to each beaker, placed on the tile and evaporated to 1-2 mL. The solutions are transferred to 10 mL plastic tubes and brought to the mark with distilled water.

The mobile forms of elements were extracted with NH_4_Ac (acetate-ammonium buffer) pH 4.8 at a soil to solution ratio of 1:10 (5 g soil, 50 mL solution) [9]. The tubes were shaken on a rotator for one hour, then centrifuged and filtered through a blue ribbon paper filter [10].

Plant samples (0.5 g) were digested in a mixture of nitric acid and distilled water (8 mL of 65% HNO_3_ + 2 mL of H_2_O) in an ETHOS EASY microwave sample digestion system (Milestone, Italy). The digestion program included four steps: 1) temperature 80 °C, time 5 min, power 800 W; 2) temperature 145 °C, time 4 min, power 1500 W; 3) temperature 195 °C, time 6 min, power 1800 W; 4) temperature 195 °C, time 10 min, power 1500 W. The obtained solutions were transferred into 10 mL test tubes.

All obtained solutions (after acid digestion of soil, extraction of mobile forms of elements, acid digestion of plant samples) were analyzed by ICP-OES. We used an ICP-OES 5800 spectrometer (Agilent Technologies, USA) with easy-fit 1.8 mm torch one piece for 5000 Series VDV/SVDV ICP-OES with a 1.8 mm ID injector with extended outer tube with slot for radial viewing (Agilent Technologies, USA), a double-pass glass cyclonic spray chamber (Agilent Technologies), an OneNeb nebulizer (Agilent Technologies, USA), and a Trident Internal Standard Kit (Glass Expansion). Samples were introduced manually. A peristaltic pump used the white/white poly-vinyl chloride pump tube for feeding and the blue/blue one for drain. V solution (100 mg/L) internal standard was added online (white/white poly-vinyl chloride pump tube) to increase the accuracy of measurements. Conditions of ICP–OES measurements are presented in Table 3. Three replicates of signal accumulation were made for each sample. Results were collected and processed by ICP Expert software 7.5.3 (Agilent Technologies). All lines were measured simultaneously. Linear functions were used for the calibration. The position and baseline correction were made for all the peaks of uncommon elements using the tools of the spectrometer software.

**Table 3:** Conditions of ICP-OES measurements.

Conditions for all lines registrations	
RF power (kW)	1.05
Plasma flow (L/min)	12.0
Axial flow (L/min)	0.7
Nebulizer flow (L/min)	1.0
Replicate read time (s)	5
Instrument stabilization delay (s)	20
Replicates	3
Sample introduction settings	
Sample uptake delay (s)	10
Pump rate (rpm)	12

Deionized water (18.2 MΩ × cm from a Simplicity system, Millipore, France) was used for the preparation of all the solutions and washing. A rare earth standard solution CCS-1-125ML (Inorganic Ventures) was used for calibration in the range of 10–1000 µg/L. The standard solution of the other metals was multi-element ICP-standard-solution IV (Carl Roth GmbH + Co. KG, Germany), calibration range 10–10000 µg/L. The standard solution of phosphorus 10000 µg/mL by High-Purity standards (USA) was used for calibration in range 0.1–100 mg/L. An internal standard solution of V (100 mg/L) was prepared from vanadyl sulphate (Sverdlovsky Zavod Khimicheskikh Reaktivov, USSR).

## Limitations

None.

## Ethics Statement

The work did not involve the use of human subjects, animal experiments and data collected from social media platforms.

## CRediT authorship contribution statement

**A.D. Kotelnikova:** Writing – original draft, Methodology, Investigation, Data curation, Visualization. **T.I. Borisochkina:** Conceptualization, Investigation. **K.A. Kolchanova:** Investigation, Data curation. **M.A. Shishkin:** Investigation, Data curation. **F.S. Egorov:** Investigation, Data curation. **V.V. Okorkov:** Resources. **O.B. Rogova:** Supervision, Project administration.

## Data Availability

Dataset on elemental composition of soils and plants under long-term application of mineral and organic fertilizers on gray forest soils in Vladimir region, Russia (Original data) (Mendeley Data)
